# Phylogenetic position of the Atlantic Gnomefish, *Scombrops oculatus* (Teleostei: Scombropidae), within the genus *Scombrops*, inferred from the sequences of complete mitochondrial genome and cytochrome *c* oxidase subunit I genes

**DOI:** 10.1080/23802359.2021.1971118

**Published:** 2021-09-06

**Authors:** Masaya Sato, Satoshi Kawato, Hikaru Oyama, Gen Kaneko, Eric J. Post, Rei Suo, Noriyuki Takai, Haruo Sugita, Hidehiro Kondo, Ikuo Hirono, Shiro Itoi

**Affiliations:** a Department of Marine Science and Resources, Nihon University, Fujisawa, Kanagawa, Japan; b Laboratory of Genome Science, Tokyo University of Marine Science and Technology, Minato, Tokyo, Japan; c School of Arts and Sciences, University of Houston-Victoria, Victoria, TX, USA; d Florida Fish and Wildlife Conservation Commission, Fish and Wildlife Research Institute, St. Petersburg, FL, USA

**Keywords:** Fish, Florida, MtDNA, Scombropid, Teleost

## Abstract

We determined the complete mitochondrial genome of the Atlantic Gnomefish, *Scombrops oculatus* (Scombropidae). The total length of mitochondrial DNA (mtDNA) was 16,515 bp and included 13 protein-coding genes, two ribosomal RNA genes, 22 transfer RNA genes, and one control region. The gene arrangement of *S. oculatus* was identical to those of three Japanese scombropid species and those of other teleosts. The phylogenetic analysis using the whole mtDNA, excluding the control region, indicates the Atlantic species is distinct from the Japanese clade, whereas that using cytochrome *c* oxidase subunit I gene showed the Atlantic species is most closely related to the African species.

The Atlantic Gnomefish, *Scombrops oculatus* (Poey 1860), is one of five species in *Scombrops*, which is the monotypic genus in the family Scombropidae (Itoi et al. 2018, 2020; Oyama et al. 2019). Although *S. oculatus* is speculated to be distributed worldwide in tropical regions based on their morphological similarities (Robins and Ray 1986), several reports indicate that this species is confined to the localities described below. The scombropid fishes are classified into *Scombrops boops* (Houttuyn 1782 ), *Scombrops gilberti* (Jordan & Snyder 1901), and *Scombrops* sp. in the northwestern Pacific Ocean (Yasuda et al. 1971; Mochizuki 1979, 1984; Shao 1987; Itoi et al. 2008, 2010, 2011, 2018, 2020), *Scombrops dubius* Gilchrist 1922 in the southwestern Indian Ocean (Heemstra 1986; Oyama et al. 2019), and *S. oculatus* in the western Atlantic Ocean, including the Caribbean Sea (Poey 1860). In these, no genetic information of the Atlantic Gnomefish, *S. oculatus*, has been available. Here, we sequenced the whole mitochondrial genome of *S. oculatus* and inferred its phylogenetic relationship among scombropid species.

The specimen of *S. oculatus* was collected on 23 July 1999 at 363–424 m depth, 43 nautical miles due east of Jupiter, Palm Beach Country, Florida, USA (26°56’N, 79°35’W), via hook-and-line, and stored in the Ichthyology Collection of the Florida Fish and Wildlife Conservation Commission, Fish and Wildlife Research Institute (https://myfwc.com/research, Eric Post, Eric.Post@MyFWC.com) under catalog number FSBC 19124 (Ruiz-Carus et al. 2003). Total genomic DNA was extracted from a scale using QIAamp FFPE Tissue Kit (Qiagen), and libraries were prepared using Nextera XT DNA Library Prep Kit (Illumina). Next-generation sequencing (NGS) was performed using MiSeq (Illumina), and sequences were assembled by SPAdes v3.14.1 (Bankevich et al. 2012) after trimming raw reads using fastp v0.20.1 (Chen et al. 2018). Several parts that could not be determined by NGS were sequenced by the Sanger method with a 3130*xl* Genetic Analyzer (Applied Biosystems). Gene annotation, including ribosomal RNA (rRNA) estimation and transfer RNA (tRNA) prediction, was conducted using MitoFish (http://mitofish.aori.u-tokyo.ac.jp/; Iwasaki et al. 2013) and Mitos WebServer (http://mitos.bioinf.uni-leipzig.de/index.py; Bernt et al. 2013), and the results of this annotation were manually verified using BLAST searches (http://blast.ncbi.nlm.nih.gov/; Altschul et al. 1997). The phylogenetic tree was constructed via the maximum likelihood method using MEGA X ver. 10.2.2 (Kumar et al. 2018).

The total mitochondrial DNA (mtDNA) length of the Atlantic Gnomefish was 16,515 bp composed of 27.92% adenine, 29.59% cytosine, 26.03% thymine and 16.46% guanine. In this sequence, the heavy-strand included 12 protein-coding genes, two rRNA genes, 14 tRNA genes and one control region, whereas the light-strand contained the remaining genes including one protein-coding gene and eight tRNA genes (DDBJ accession number LC603186). The arrangement of the mitochondrial genes was identical to that of three Japanese scombropids (Tsunashima, Itoi et al. 2016; Tsunashima, Yamada et al. 2016; Mochizuki et al. 2017) and also to those of other teleosts (Miya et al. 2003). Each gene in the Atlantic Gnomefish mtDNA had the same start and stop codons as the Japanese scombropids. The locations of the control region in *S. oculatus* mtDNA are between the tRNA genes *tRNA-Pro* and *tRNA-Phe*. Additionally, the two rRNA genes *12S rRNA* and *16S rRNA* are located between *tRNA-Phe* and *tRNA-Leu(UAA)*, separated by *tRNA-Val*. The mtDNA sequence of the Atlantic Gnomefish showed 94.2–94.5% identity with those of these Japanese scombropids (Tsunashima, Itoi et al. 2016; Tsunashima, Yamada et al. 2016; Mochizuki et al. 2017).

We constructed a phylogenetic tree by maximum likelihood method with the GTR + G + I substitution model using whole mitochondrial genome sequences from 29 teleost species, supporting the monophyly of Scombropidae, and showed that a clade of the Atlantic Gnomefish, *S. oculatus*, was distinct from that including the Japanese scombropids, *S. boops*, *S. gilberti* and *Scombrops* sp. (Figure 1(a)). In addition, a maximum likelihood tree with the HKY + I substitution model using partial *COI* sequences from 13 teleost species shows that the Atlantic Gnomefish is most closely related to the African Gnomefish, *S. dubius* (Figure 1(b)), indicating that a common ancestor of the scombropid species diverged into the common ancestors of the Atlantic/African scombropids and the Japanese scombropids followed by further species divergence. Assuming an average 1.5–2.5% sequence divergence per million years, as inferred from the generally accepted molecular clock for cytochrome *b* in fishes (Bernardi et al. 2004), the difference in the nucleotide sequences between the Atlantic and Japanese species clades was approximately 7% corresponding to 2.8–4.7 million years ago (mya). The divergence timing was close to the latest closure of the Isthmus of Panama estimate (approximately 3.0 mya; O’Dea et al. 2016). These results suggest that the common ancestor of scombropid species might have been divided into the Pacific and Atlantic/African clades by the formation of the Isthmus of Panama.

**Figure 1.**
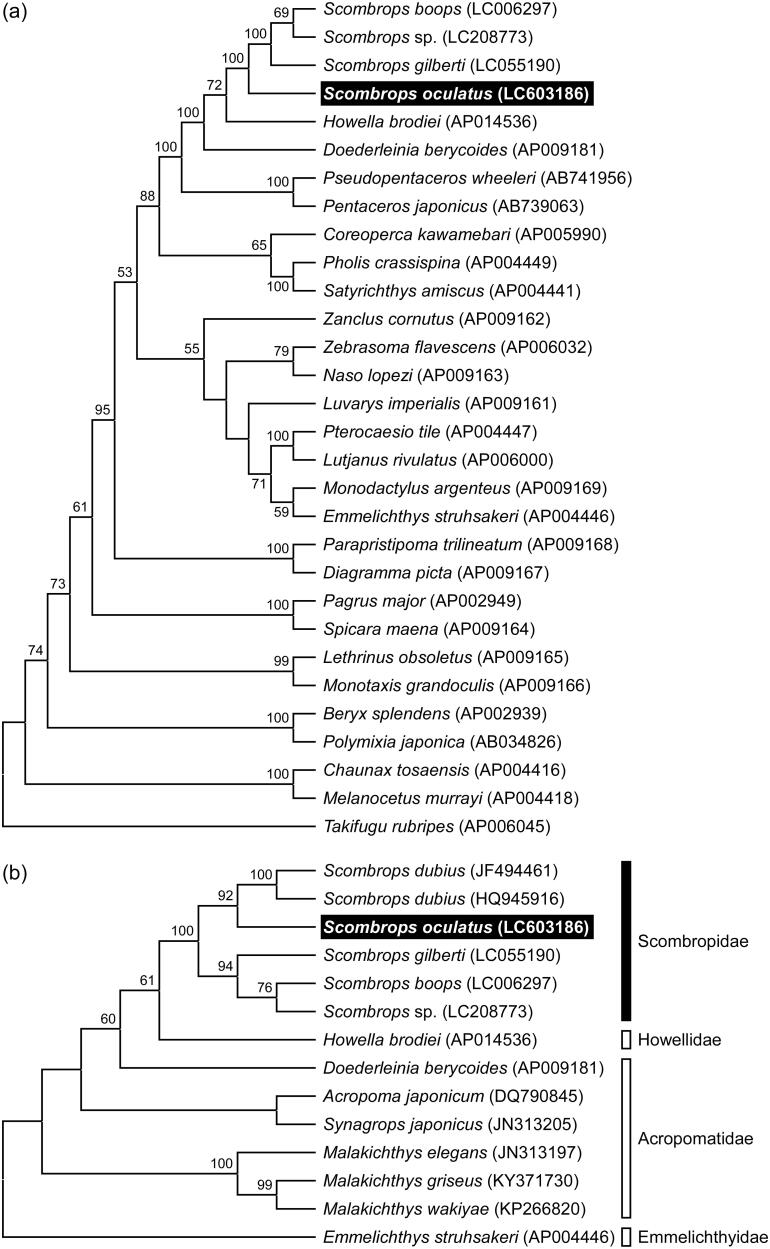
Phylogenetic relationship of the Atlantic Gnomefish *Scombrops oculatus* in related teleosts inferred from (a) whole mitochondrial genome excluding the control region and (b) partial sequence of *COI* gene. Tree was generated by maximum likelihood analysis under the nucleotide substitution models GTR + G + I for whole mitochondrial genome and HKY + I for *COI* gene. Numbers at branches denote the bootstrap percentages from 1000 replicates. Only bootstrap values exceeding 50% are presented. LC603186 in parentheses indicates the accession number deposited in the DDBJ/EMBL/GenBank databases in this study and accession numbers for reference sequences are shown in parentheses. The sequences of *Takifugu rubripes* and *Emmelichthys struhsakeri* are used as the outgroups for trees of whole mitochondrial genome and *COI* gene, respectively.

Thus, the characterization of the *S. oculatus* mtDNA proved to be useful for understanding the phylogeny and taxonomy of the Scombropidae, and for investigating the ecology of these fishes.

## Data Availability

The genome sequence data that support the findings of this study are openly available in GenBank of NCBI at https://www.ncbi.nlm.nih.gov/ under the accession no. LC603186. The associated BioProject, SRA, and Bio-Sample numbers are PRJDB12050, DRA012467, and SAMD00394227, respectively.

## References

[CIT0001] Altschul SF, Madden TL, Schaffer AA, Zhang J, Zhang Z, Miller W, Lipman DJ. 1997. Gapped BLAST and PSI-BLAST: a new generation of protein database search programs. Nucleic Acids Res. 25(17):3389–3402. 9254694 10.1093/nar/25.17.3389 PMC146917

[CIT0002] Bankevich A, Nurk S, Antipov D, Gurevich AA, Dvorkin M, Kulikov AS, Lesin VM, Nikolenko SI, Pham S, Prjibelski AD, et al. 2012. SPAdes: a new genome assembly algorithm and its applications to single-cell sequencing. J Comput Biol. 19(5):455–477. 22506599 10.1089/cmb.2012.0021 PMC3342519

[CIT0003] Bernardi G, Bucciarelli G, Costagliola D, Robertson DR, Heiser JB. 2004. Evolution of coral reef fish *Thalassoma* spp. (Labridae). 1. Molecular phylogeny and biogeography. Mar Biol. 144(2):369–375.

[CIT0004] Bernt M, Donath A, Jühling F, Externbrink F, Florentz C, Fritzsch G, Pütz J, Middendorf M, Stadler PF. 2013. MITOS: improved *de novo* metazoan mitochondrial genome annotation. Mol Phylogenet Evol. 69(2):313–319. 22982435 10.1016/j.ympev.2012.08.023

[CIT0005] Chen S, Zhou Y, Chen Y, Gu J. 2018. Fastp: an ultra-fast all-in-one FASTQ preprocessor. Bioinformatics. 34(17):i884–i890. 30423086 10.1093/bioinformatics/bty560 PMC6129281

[CIT0006] Heemstra PC. 1986. Family No. 177: scombropidae. In: Smith MM, Heemstra PC, editors. Smiths’ sea fishes. Berlin: Springer-Verlag; p. 563.

[CIT0007] Itoi S, Mochizuki Y, Tanaka M, Oyama H, Tsunashima T, Yamada R, Shishido H, Masuda Y, Nakai S, Takai N, et al. 2018. Species composition of the genus *Scombrops* (Teleostei, Scombropidae) in the waters around the Japanese Archipelago: detection of a cryptic species. Mitochondr DNA A. 29(8):1293–1300. 10.1080/24701394.2018.1445243 29488422

[CIT0008] Itoi S, Odaka J, Noguchi S, Noda T, Yuasa K, Muraki T, Tanabe T, Takai N, Yoshihara K, Sugita H. 2011. Genetic homogeneity between adult and juvenile populations of *Scombrops gilberti* (Percoid, Scombropidae) in the Pacific Ocean off the Japanese Islands. Fish Sci. 77(6):975–981.

[CIT0009] Itoi S, Odaka J, Yuasa K, Akeno S, Nakajima A, Suenaga A, Noda T, Akimoto S, Myojin T, Ikeda Y, et al. 2010. Distribution and species composition of juvenile and adult scombropids (Teleostei, Scombropidae) in Japanese coastal waters. J Fish Biol. 76(2):369–378. 20738713 10.1111/j.1095-8649.2009.02493.x

[CIT0010] Itoi S, Takai N, Naya S, Dairiki K, Yamada A, Akimoto S, Yoshihara K, Sugita H. 2008. A species identification method for *Scombrops boops* and *Scombrops gilberti* based on polymerase chain reaction-restriction fragment length polymorphism analysis of mitochondrial DNA. Fish Sci. 74(3):503–510.

[CIT0011] Itoi S, Tanaka M, Mochizuki Y, Oyama H, Togawa S, Yamada R, Ito T, Shishido H, Masuda Y, Abe K, et al. 2020. Tandem PCR-RFLP analysis helps distinguish among three Japanese gnomefish (Teleostei: Scombropidae: *Scombrops*). Ichthyol Res. 67(1):197–202.

[CIT0012] Iwasaki W, Fukunaga T, Isagozawa R, Yamada K, Maeda Y, Satoh TP, Sado T, Mabuchi K, Takeshima H, Miya M, et al. 2013. MitoFish and MitoAnnotator: a mitochondrial genome database of fish with an accurate and automatic annotation pipeline. Mol Biol Evol. 30(11):2531–2540. 23955518 10.1093/molbev/mst141 PMC3808866

[CIT0013] Kumar S, Stecher G, Li M, Knyaz C, Tamura K. 2018. MEGA X: molecular evolutionary genetics analysis across computing platforms. Mol Biol Evol. 35(6):1547–1549. 29722887 10.1093/molbev/msy096 PMC5967553

[CIT0014] Miya M, Takeshima H, Endo H, Ishiguro NB, Inoue JG, Mukai T, Satoh TP, Yamaguchi M, Kawaguchi A, Mabuchi K, et al. 2003. Major patterns of higher teleostean phylogenies: a new perspective based on 100 complete mitochondrial DNA sequences. Mol Phylogenet Evol. 26(1):121–138. 12470944 10.1016/s1055-7903(02)00332-9

[CIT0015] Mochizuki K. 1979. Age and growth of the two Japanese scombropids, *Scombrops boops* and *S. gilberti*. Jpn J Ichthyol. 26:62–68.

[CIT0016] Mochizuki K. 1984. Family scombropidae. In: Masuda H, Amaoka K, Araga C, Uyeno T, Yoshino T, editors. The fishes of the Japanese Archipelago. Tokyo: Tokai University Press; p. 152.

[CIT0017] Mochizuki Y, Yamada R, Shishido H, Masuda Y, Nakai S, Takai N, Itoi S, Sugita H. 2017. Complete mitochondrial genome of an undescribed gnomefish of the genus *Scombrops* (Teleostei, Scombropidae) from southern waters off Kyushu Island, Japan. Mitochondr DNA B. 2(1):106–108. 10.1080/23802359.2017.1289348 PMC7799919 33473732

[CIT0018] O’Dea A, Lessios HA, Coates AG, Eytan RI, Restrepo-Moreno SA, Cione AL, Collins LS, de Queiroz A, Farris DW, Norris RD, et al. 2016. Formation of the Isthmus of Panama. Sci Adv. 2(8):e1600883. 27540590 10.1126/sciadv.1600883 PMC4988774

[CIT0019] Oyama H, Itoi S, Ueda H, Mochizuki Y, Tanaka M, Ito T, Shishido H, Masuda Y, Takai N, Sugita H. 2019. Genetic difference between African and Japanese scombropid populations based on cytochrome *c* oxidase subunit I gene sequences. Mitochondr DNA B. 4(1):1016–1020.

[CIT0020] Poey F. 1860. Poissons de Cuba. Mem Hist Nat Isla Cuba. 2:115–356.

[CIT0021] Robins CR, Ray GC. 1986. A Field Guide to Atlantic Coast Fishes: North America (Peterson Field Guides No. 32). Boston, New York: Houghton Mifflin Co.; p. 156.

[CIT0022] Ruiz-Carus R, Matheson RE, Jr, Vose FE. 2003. First record of the escolar chino, *Scombrops oculatus* (Poey, 1860) off the U.S. Atlantic coast, with comments on its taxonomy. Florida Scientist. 66:287–290.

[CIT0023] Shao K-T. 1987. First record of *Scombropidae* (Pisces: Percoidei) from Taiwan. Bull Inst Zool Academia Sinica. 26:191–194.

[CIT0024] Tsunashima T, Itoi S, Abe K, Takigawa T, Inoue S, Kozen T, Ono N, Noguchi S, Nakai S, Takai N, et al. 2016. The complete mitochondrial genome of the gnomefish *Scombrops boops* (Teleostei, Perciformes, Scombropidae) from the Pacific Ocean off the Japanese Islands. Mitochondr DNA Part A. 27(1):785–786. 10.3109/19401736.2014.987242 25484172

[CIT0025] Tsunashima T, Yamada R, Abe K, Noguchi S, Itoi S, Nakai S, Takai N, Sugita H. 2016. Phylogenetic position of Scombropidae within teleostei: the complete mitochondrial genome of the gnomefish, *Scombrops gilberti*. Mitochondr DNA Part A. 27(5):3446–3448. 10.3109/19401736.2015.1063135 26153741

[CIT0026] Yasuda F, Mochizuki K, Kawajiri M, Nose Y. 1971. On the meristic and morphometric differences between *Scombrops boops* and *S. gilberti*. Jpn J Ichthyol. 18:118–124.

